# Standard of care versus new-wave corticosteroids in the treatment of Duchenne muscular dystrophy: Can we do better?

**DOI:** 10.1186/s13023-021-01758-9

**Published:** 2021-03-04

**Authors:** Stephanie Kourakis, Cara A. Timpani, Dean G. Campelj, Patricia Hafner, Nuri Gueven, Dirk Fischer, Emma Rybalka

**Affiliations:** 1 Institute for Health and Sport (IHeS), Victoria University, Melbourne, VIC Australia; 2 Australian Institute for Musculoskeletal Science (AIMSS), St Albans, VIC Australia; 3 Division of Neuropediatrics and Developmental Medicine, University Children’s Hospital of Basel (UKBB), Basel, Switzerland; 4 School of Pharmacy and Pharmacology, University of Tasmania, Hobart, TAS Australia

**Keywords:** Duchenne muscular dystrophy, Corticosteroids, Standard of care, Anti-inflammatory drugs, Anti-inflammation

## Abstract

**Background:**

Pharmacological corticosteroid therapy is the standard of care in Duchenne Muscular Dystrophy (DMD) that aims to control symptoms and slow disease progression through potent anti-inflammatory action. However, a major concern is the significant adverse effects associated with long term-use.

**Main:**

This review discusses the pros and cons of standard of care treatment for DMD and compares it to novel data generated with the new-wave dissociative corticosteroid, vamorolone. The current status of experimental anti-inflammatory pharmaceuticals is also reviewed, with insights regarding alternative drugs that could provide therapeutic advantage.

**Conclusions:**

Although novel dissociative steroids may be superior substitutes to corticosteroids, other potential therapeutics should be explored. Repurposing or developing novel pharmacological therapies capable of addressing the many pathogenic features of DMD in addition to anti-inflammation could elicit greater therapeutic advantages.

## Background

Pharmacological corticosteroid therapy is the standard of care in Duchenne Muscular Dystrophy (DMD), a progressive, genetically inherited neuromuscular diseases arising from mutations in the dystrophin gene. Null dystrophin protein expression compromises the stability and permeability of the sarcolemma and initiates chronic muscle damage, inflammation, degeneration and wasting, with death as an eventual outcome due to cardiorespiratory failure. With no cure, corticosteroid treatment aims to control symptoms and slow disease progression through potent anti-inflammatory action. Although the clinical efficacy and short-term benefits of steroid use is established, of major concern are the significant adverse effects associated with long term-use. Although alternatives such as dissociative steroids are developed, non-steroidal therapeutics with favourable side-effect profiles that can be rapidly translated into a clinical setting should also be investigated as alternatives to address the high unmet medical need in the treatment of DMD patients.

## Introduction

Duchenne muscular dystrophy (DMD) is a X-linked recessive disorder that arises from mutations in the dystrophin gene causing absent or truncated dystrophin protein [1]. Approximately 60% of mutations arise from large deletion or insertion frameshift errors and 40% arise from small frameshift errors or point mutations [1]. Out of frame mutations usually result in the complete ablation of dystrophin protein expression (DMD) while in frame mutations usually result in partial dystrophin expression and Becker muscular dystrophy (BMD), a milder form of dystrophinopathy [2]. Designated a rare disease, DMD affects 1 in 3500–7000 live male births worldwide [3, 4]. It is characterised by inflammation and progressive degeneration of skeletal and cardiac muscles [1, 5]. The deterioration of ambulatory function arises initially during childhood and culminates in complete loss by early adolescence [6]. Further complications include scoliosis, contractures and cardiorespiratory decline, which over time contribute to death in early adulthood [4]. Therapies that target the underlying genetic mutations of DMD such as human micro-dystrophin gene delivery [7, 8] and antisense oligonucleotide exon skipping therapeutics [9, 10] offer a new avenue for a potential cure. However, long term clinical benefit has yet to be established for either. Presently, standard of care is corticosteroids (glucocorticoids), which aim to delay progression of the disease by reducing inflammation-induced muscle damage and thus muscle strength loss and disease progression [11].

**Fig. 1:**
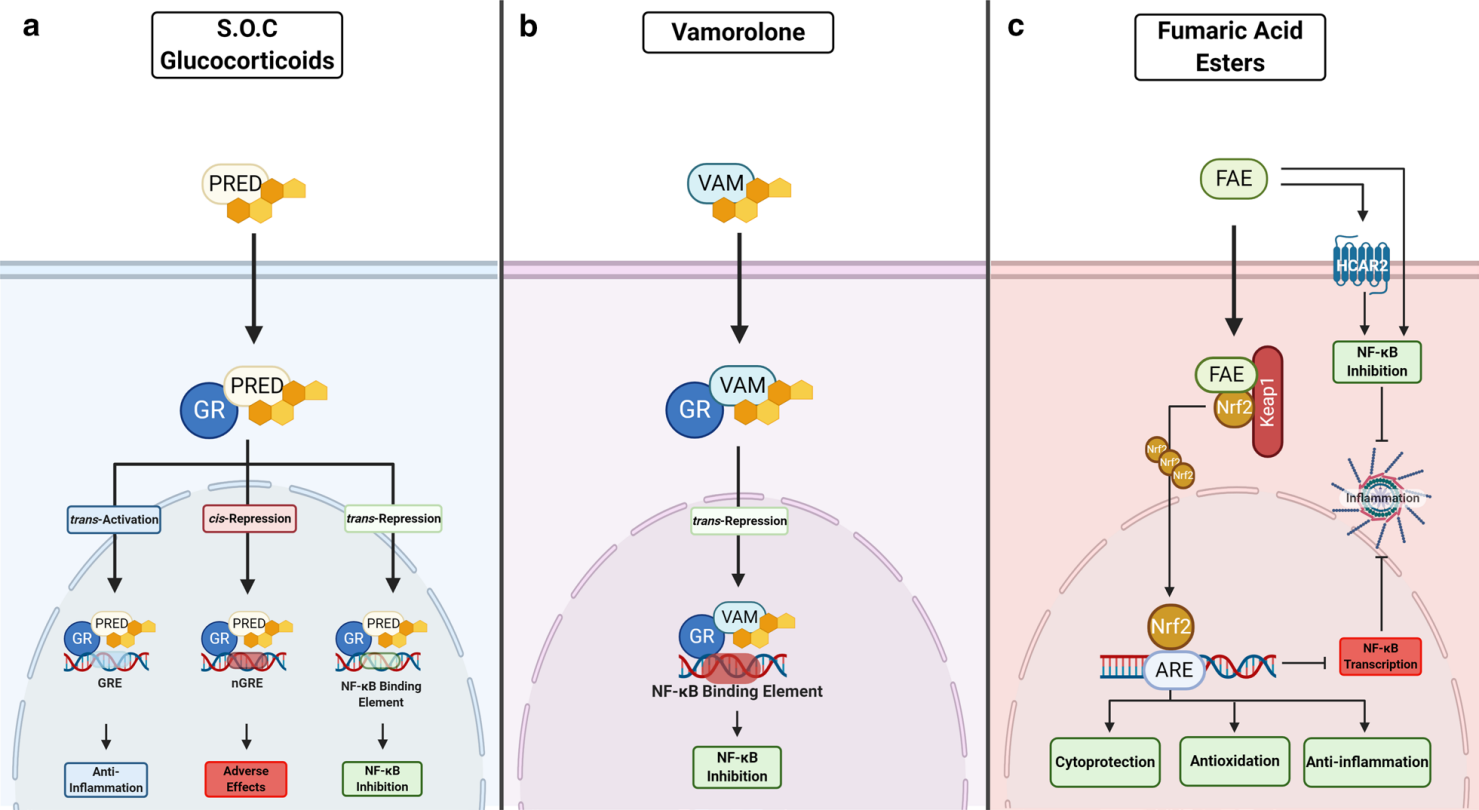
*Comparing the mechanisms of action of standard of care (S.O.C) glucocorticoids (i.e., prednisone and deflazacort) with novel dissociative steroid vamorolone and fumaric acid esters (FAE).* **a** Glucocorticoids like prednisone (PRED), diffuse through the cell membrane, bind to the cytoplasmic nuclear hormone receptor (glucocorticoid receptor (GR)) to form a receptor-ligand complex, which translocates to the nucleus. This complex indirectly binds to the glucocorticoid response element (GRE), activating target genes that are associated with broad spectrum anti-inflammation (trans-activation), as well as the nuclear factor kappa B (NF-κB) binding element to supress transcription of master inflammatory regulator, NF-κB (trans-repression). These mechanisms elicit the beneficial effects of glucocorticoids in DMD. In contrast, adverse effects are mediated through direct binding of the GR-ligand complex to negative GRE on other target genes, which represses their transcription (*cis*-repression). **b** Similarly, vamorolone binds to the GR and retains the anti-inflammatory effects characteristic of standard of care glucocorticoids, inducing transrepression with hardly any transactivation or *cis*-repression to elicit fewer adverse effects. **c** Therapeutic efficacy of FAEs is mediated through the dual activation of the nuclear factor erythroid 2-related factor 2 (Nrf2) transcriptional pathway and hydroxycarboxylic acid receptor 2 (HCAR2). Nrf2 regulates the essential cellular defence system when electrophiles/FAE bind and disrupt the interaction between Nrf2 and its negative repressor (Kelch-like ECH-associated protein 1 (Keap1)). This disruption allows Nrf2 to translocate to the nucleus, bind to the antioxidant response element (ARE) resulting in cytoprotection. Nrf2 and HCAR2 both strongly inhibit NF-κB signalling within the cellular inflammatory response. Created with BioRender.com

Glucocorticoids diffuse through the cell membrane, binding to the cytoplasmic nuclear hormone receptor (glucocorticoid receptor (GR)) to form a receptor-ligand complex, which translocates to the nucleus [12, 13]. The GR supresses the pro-inflammatory nuclear factor kappa B (NF-κB) signalling pathway, to exert the well-known potent anti-inflammatory effects of steroids in a process termed transrepression [14, 15] (Fig. 1). NF-κB transcriptional activity is chronically elevated in DMD and is recognised as a key molecular feature of disease onset and progression [16, 17]. As well as strong transrepressor activity against NF-κB, the receptor-ligand complex also directly binds the glucocorticoid response element (GRE) to increase the transcription of target genes (e.g. nuclear factor of kappa light polypeptide gene enhancer in B-cells inhibitor, alpha (IκBα) a protein inhibitor of NF-κB, annexin 1, interleukin-10 (IL-10)) to elicit broad spectrum anti-inflammatory action in a process termed transactivation [15, 18] (Fig. 1). However, the GR receptor-ligand complex can also directly bind negative GRE sites (nGRE) on other target genes (e.g., corticotrophin-releasing hormone (CRH), osteocalcin (OC), proopiomelanocortin (POMC)) to supress gene transcription in a process called *cis*-repression [19, 20]. GR-mediated *cis*-repression is associated with the notorious adverse effects elicited by glucocorticoids (Fig. 1) including growth retardation/failure to thrive (CRH and POMC), osteoporosis (OC) and skin fragility (keratins) (for detailed reviews see [19, 20]). Although glucocorticoids are routinely prescribed for DMD patients, questions still remain as to whether some of the more severe side effects (e.g., excessive weight gain, cataracts, behavioural issues, delayed growth and osteoporosis) contraindicate the intended benefits. This review compares the mechanism of action of commonly used and novel glucocorticoids together with their adverse effects, against pharmacological alternatives that may offer superior therapeutic benefit.

## Standard of Care in DMD

Corticosteroids are a class of steroid hormones that are released by the adrenal cortex, which include glucocorticoids [21]. Glucocorticoids bind to and activate the GR [12], regulating several physiological processes including immune response [22, 23], metabolism [24], mood and cognitive function [25]. Pharmacological analogues (e.g. prednisone and dexamethasone) are often prescribed for auto-immune and inflammatory diseases because of their considerable immuno-modulatory properties [23, 26] and have become a clinical mainstay, especially in the treatment of DMD. However, the therapeutic benefits of glucocorticoids are limited by several adverse effects associated with their long-term use.

The glucocorticoids prednisone/prednisolone and deflazacort are the gold standard of care for the treatment of DMD [27]. Prednisone is a synthetic, anti-inflammatory glucocorticoid, which is converted to prednisolone in the liver [28]. Prednisone was approved by the Food and Drug Administration (FDA) in the 1950s and is prescribed as a dual immunosuppressive and anti-inflammatory agent to treat a broad range of conditions including, but not limited to, rheumatic [26, 29, 30], dermatologic [31], ophthalmic [32, 33], respiratory [34, 35], hematologic [36] and gastrointestinal [37, 38] indications. Prednisone/prednisolone is currently used off-label for DMD to slow progressive muscle weakness and delay associated disease milestones (e.g. Gowers’ manoeuvre, loss of ambulation and nocturnal ventilation) [39, 40].

In DMD, the absence of dystrophin causes muscle fibres to become vulnerable to contraction-induced damage prompting them to undergo repeated cycles of necrosis and regeneration until muscle mass is progressively replaced by fibrous connective tissue and fat resulting in muscle weakness and loss of function [41]. It was suggested that prednisone slows progression of muscle weakness [40, 42–44] and in doing so prolongs ambulation for 2–3 years [45–47] and improves pulmonary function [48, 49]. This function seems particularly relevant to early intervention i.e. from 2–4 years of age [50]. Unfortunately, high dosage or long-term use of prednisone is typically accompanied by mild to severe adverse effects that can impact the quality of life, reduce patient adherence and limit overall therapeutic outcomes in DMD sufferers. These include: excessive weight gain [40, 42, 48, 49, 51], adrenal insufficiency [52], stunted growth [51], cushingoid appearance [48, 49], behavioural changes [53], decreased bone mineral density [54] and increased incidence of fractures [46, 55]. These adverse effects, in combination with the already progressive, terminal nature of DMD may further place undue strain on patients and their families, and in particular, increase parental stress. For this reason, short-term intermittent prednisone treatment has been investigated as an alternative to chronic therapy, with noticeably reduced adverse effects and family stress and no impact on therapeutic activity [40].

Deflazacort, an oxazoline derivative of prednisolone [56] was approved in 2017 for DMD patients aged 5 years and older. Similar to prednisone, deflazacort is also used to treat a variety of other diseases based on its anti-inflammatory and immunosuppressive effects. Deflazacort shows comparable efficacy to prednisone in DMD patients but has been associated with improved outcomes such as greater delay in loss of ambulation [46, 47, 57–59], cardiac, pulmonary and motor function [46, 58–60] and a lower risk of scoliosis [46, 47] in contrast to prednisone/prednisolone [59]. Deflazacort is purported as a steroid alternative with fewer adverse effects and possibly with less risk of weight gain compared to prednisone [46, 47, 58, 61]. However, in comparison to prednisone it is associated with behavioural changes [53] and cataract formation [46, 47, 57, 58]. The effects of deflazacort on development and bone health have been inconsistent. Balaban (2005), Marden (2020), Bello (2015) and Biggar (2001) associated deflazacort with higher frequencies of growth delay and bone fractures [46, 47, 57, 58] whereas Mesa (1991) and Angelini (1994) reported fewer effects on bone mass and fractures [60, 62] in comparison to prednisone, making it unclear how deflazacort affects bone health.

The cardiac effects of corticosteroid treatment in DMD patients are not well characterised, although their use was associated with benefits such as improved overall cardiac function and delayed onset of cardiomyopathy [63–65]. Conversely, other studies indicated that patients who commence corticosteroid treatment at an early age (< 5 years old) are more likely to develop premature onset of cardiomyopathy compared to those who initiate treatment in later childhood as well as those who do not undergo treatment [66]. Long-term administration of glucocorticoids has also been associated with acceleration of protein breakdown and inhibition of protein synthesis [67–69], which may ultimately lead to skeletal muscle atrophy [70–72]. These catabolic effects appear to be mediated, at least in part, via modulation of insulin-like growth factor-1 (IGF-1) signalling and pro-atrophy signalling through Atrogin-1 [69, 73]. Despite atrophy being counterintuitive to the attenuation of muscle wasting, glucocorticoids are still able to ablate some clinical symptoms through anti-inflammatory function. While these studies contributed to our understanding concerning the impact of corticosteroid treatment on DMD patients, some are disadvantaged by their short duration and follow-up times highlighting the need for longitudinal studies to understand the full impact of long-term corticosteroid use. Although there is ongoing research to establish the most effective dose and regimens for glucocorticoids, safer alternatives are needed that offer a better benefit to side effect profile. In this regard, a novel dissociative steroid has recently shown some promise.

### Vamorolone: a novel dissociative steroid

Vamorolone (formerly VBP-15), is a first-in-class anti-inflammatory steroid analogue [74] that is currently being investigated as a replacement for traditional standard of care glucocorticoids in DMD. The structure of vamorolone is similar to other glucocorticoids: it binds to the GR and retains the anti-inflammatory effects characteristic of traditional steroids, preferentially inducing transrepression with little-to-no transactivation or *cis*-repression (Fig. 1). As such, it is purported to elicit fewer adverse effects [13, 75]. Vamorolone is also a mineralocorticoid receptor (MR) antagonist [18], and thus has the potential to treat DMD-associated cardiomyopathy through modulation of blood pressure. Dystrophin-deficient hearts are especially sensitive to damage facilitated through MR activation [18] although specific MR antagonists (e.g., spirololactone) showed no pre-clinical efficacy when administered with an angiotensin converting enzyme inhibitor drug in *mdx* mice with exacerbated disease [76]. The development of vamorolone for DMD was initiated with a Phase I clinical trial (NCT02415439) in healthy volunteers to assess its safety, tolerability and pharmacokinetics. Vamorolone was well-tolerated at all dose levels, with pharmacokinetic and metabolic profiles similar to that of prednisone but without the associated adverse effects and safety concerns of traditional glucocorticoids (e.g., dexamethasone and prednisone) [77]. Subsequently, a Phase IIa trial in DMD boys aged 4 to < 7 years (NCT02760277) investigated safety and tolerability of a range of vamorolone doses and explored potential efficacy over 6 months. Similar to the Phase I trial, Vamorolone was reported to be safe and well-tolerated and met the primary efficacy outcome of improved muscle function without evidence of adverse effects [78, 79]. Rather than transition back to glucocorticoids, all patients included in the study requested to continue treatment on vamorolone under the 24-month long-term open-label extension study (NCT03038399), which was recently completed [80]. In this study, treatment with vamorolone was associated with improvements in some motor outcomes and a favourable safety profile as fewer adverse effects were reported (less incidence of weight gain, behavioural changes and cushingoid appearance) [80] compared to those previously reported with traditional corticosteroid use. Importantly, vamorolone, did not repress growth, which is usually observed with SOC treatment. An ongoing Phase IIb randomised, double-blind trial (NCT03439670) is designed to demonstrate efficacy and safety of vamorolone at different doses compared to prednisone and placebo over 24 weeks. Based on the available data, vamorolone received orphan drug status in the United States and Europe and will likely establish itself as a safer and superior alternative to current SOC glucocorticoids to benefit DMD patients (as well as patients diagnosed with other chronic inflammatory diseases).

### Pharmacological anti-inflammatory alternatives for DMD

Despite some advances, an unmet clinical need remains for DMD disease-modifying drugs that are well tolerated and effectively mitigate disease progression. Steroids have prevailed as the only disease modifying drugs against DMD for more than a decade, despite their propensity to promote muscular atrophy [70–72]. Presumably, their intense anti-inflammatory function is more influential to attenuate muscle wasting than their atrophic effect progresses it [81]. Particularly, chronic inflammation is a driver of fast type II muscle fibre loss, which is pronounced in DMD [82]. It is possible that other potent anti-inflammatory drugs could have equivalent, if not greater, beneficial effects on mitigating DMD without the associated side-effects—particularly those pertaining to muscle atrophy, which is clearly counterintuitive in DMD treatment.

Many anti-inflammatory drugs have been tested pre-clinically (typically in the *mdx* mouse) and while a few have recently undergone clinical testing (the covalently-linked salicylic acid and docosahexaenoic acid small molecule, edasalonexent/CAT-1004: NCT02439216, NCT03703882 [83, 84]; and the tetracosactide (cosyntrophin) formulation synthetic melanocortin receptor agonist, MNK-1411: NCT03400852 [85]), their clinical development was terminated due to lack of efficacy and/or recruitment issues. There are a variety of other potent anti-inflammatory drugs on the market that are yet to be investigated in DMD and that could be therapeutically advantageous compared to the glucocorticoids. Fumaric acid esters (FAEs) such as dimethyl fumarate (DMF), monomethyl fumarate (MMF) and diroximel fumarate (DRF) are well-known for their anti-inflammatory and immuno-modulatory effects [86–89]. FAEs are presently approved for several indications including psoriasis and Remitting-Relapsing Multiple Sclerosis (RRMS) [90–93]. These drugs display robust safety profiles and comprehensive clinical utility for diseases characterised by inflammation and oxidative stress, and both of these are notorious drivers of DMD [94–102]. The therapeutic efficacy of FAEs appear to be mediated through the dual activation of the nuclear factor erythroid 2-related factor 2 (Nrf2) transcriptional pathway [88, 103–105] and the hydroxycarboxylic acid receptor 2 (HCAR2) [106, 107] (Fig. 1). Nrf2 regulates the essential cellular defence system that counteracts potentially harmful stimuli through the upregulation of antioxidants and cytoprotective response genes [108, 109]. Both Nrf2 and HCAR2 also strongly inhibit NF-κB signalling of the cellular inflammatory response [106, 110, 111]. Over the last decade, several Nrf2 activating drugs have been developed and trialled in both clinical [112–117] and pre-clinical [118–121] settings highlighting the broad therapeutic utility of targeted Nrf2 activation against diseases associated with inflammation and oxidative stress [122]. There are many pharmacological as well as nutraceutical activators of Nrf2 including those approved for use as disease modifying treatments (DMF, MMF, DRF, ursodiol and oltipraz). Whilst no clinical trials have investigated FAEs for their dual Nrf2/HCAR2 activator action in DMD patients at present [123], the synthetic flavonone, epicatechin, which has strong anti-inflammatory properties [124, 125] and is an Nrf2 activator [126] (in addition to several other purported mechanisms of action such as myostatin suppression), has tested favourably in the *mdx* mouse [127, 128] and is in clinical development for BMD (NCT04386304) [129] (but not DMD). Given the safety and efficacy of FAEs and targeted Nrf2 activators established in other studies, further translational investigations should be undertaken to assess the therapeutic potential of novel and repurposed modulators of DMD pathology as corticosteroid alternatives. This is particularly relevant since Nrf2 activation has additional benefits beyond the anti-inflammatory activity of glucocorticoids, which include energy re-balancing through mitochondrial biogenesis as well as muscle regeneration and repair [123]. Through multiple mechanisms, FAEs can also modify a more extensive cytokine profile than glucocorticoids [130].

## Conclusion

In DMD, glucocorticoids represent the most frequently used drug class for the management of symptoms. However, the current standard of care (prednisone/prednisolone or deflazacort) acts non-selectively [131] contributing to many associated complications which impact quality of life. More recently, research has focused on novel selective, dissociative steroids, such as vamorolone [74, 77–79], which may provide a better alternative by reducing steroid-associated adverse effects. Although novel dissociative steroids may be a superior substitute to glucocorticoids, other potential therapeutics should be explored. Repurposing or developing novel pharmacological therapies that are capable of addressing the many downstream consequences of dystrophin deficiency, such as FAEs and novel Nrf2 activators, respectively, may be a viable option to improve patient quality of life without severe adverse events like those observed with corticosteroid use. Since they activate alternative receptors/signalling pathways to glucocorticoids, there is also scope for combined FAE and corticosteroid regimens that could synergistically amplify therapeutic potential.

## Data Availability

Not applicable.

## Abbreviations

DMD: Duchenne Muscular Dystrophy
GR: Glucocorticoid Receptor
NF-κB: Nuclear Factor kappa B
GRE: Glucocorticoid Response Element
Iκ-Bα: Nuclear Factor of Kappa Light Polypeptide Gene Enhancer in B-cells Inhibitor, Alpha
FDA: Food and Drug Administration
IGF-1: Insulin-like Growth Factor 1
MR: Mineralocorticoid Receptor
DHA: Docosahexaenoic Acid
FAEs: Fumaric Acid Esters
DMF: Dimethyl Fumarate
MMF: Monomethyl Fumarate
DRF: Diroximel Fumarate
RRMS: Relapsing–remitting Multiple Sclerosis
Nrf2: Nuclear factor erythroid 2-Related Factor 2
HCAR2: Hydroxycarboxylic Acid Receptor 2
